# Severe aplastic anemia patients with infection who received an allogeneic hematopoietic stem cell transplantation had a better chance: Long-term outcomes of a multicenter study

**DOI:** 10.3389/fimmu.2022.955095

**Published:** 2022-09-05

**Authors:** Limin Liu, Miao Miao, Hailong He, Shunqing Wang, Yanming Zhang, Ailian Guo, Wenjing Jiao, Meiqing Lei, Yifeng Cai, Xiaohui Shangguan, Zefa Liu, Jinge Xu, Xiaoli Li, Liansheng Zhang, Depei Wu

**Affiliations:** ^1^ National Clinical Research Center for Hematologic Diseases, Jiangsu Institute of Hematology, The First Affiliated Hospital of Soochow University, Suzhou, China; ^2^ Department of Hematology, Children’s Hospital of Soochow University, Suzhou, China; ^3^ Department of Hematology, Guangzhou First People’s Hospital, Guangzhou Medical University, Guangzhou, China; ^4^ Department of Hematology, The Affiliated Huai’an Hospital of Xuzhou Medical University and The Second People’s Hospital of Huai’an, Huai’an, China; ^5^ Department of Hematology, Xian Yang Central Hospital, Xianyang, China; ^6^ Department of Hematology in Haikou Municipal People’s Hospital, Affiliated Haikou Hospital, Haikou, China; ^7^ Department of Hematology, The Affiliated Hospital of Nantong University, Nantong, China; ^8^ Department of Hematology, Longyan First Hospital, Affiliated to Fujian Medical University, Longyan, China; ^9^ Department of Hematology, People Hospital of Xinghua, Xinghua, China; ^10^ Department of Hematology, The Second Affiliated Hospital of Xuzhou Medical University, Xuzhou, China; ^11^ Department of Hematology, Soochow Hopes Hematonosis Hospital, Suzhou, China; ^12^ Department of Hematology, The Second Hospital of Lanzhou University, Lanzhou, China

**Keywords:** Severe aplastic anemia, infection, allogeneic hematopoietic stem cell transplantation, therapy, results

## Abstract

**Background and aims:**

How to select the treatment is a challenge for the management of acquired patients with infections. This study aimed at comparing the outcomes of SAA with infections who had an allogeneic hematopoietic stem cell transplantation (allo-HSCT)with that of patients who had an infection and received non-HSCT therapy.

**Methods:**

We retrospectively compared the outcomes of patients with acquired SAA and infections who had an allo-HSCT (*n* = 141) with that of patients who had an infection and received non-HSCT therapy (n = 186) between July 2004 and January 2020.

**Results:**

The treatment-related mortality (TRM) of grade 1-2 infections in the HSCT and non-HSCT groups was 24.99% and 13.68%, respectively (*P =* 0.206), while the TRM of grade 3-4 infections was lower in the HSCT group than that observed in the non-HSCT group (18.54% vs. 33.33%, *P =* 0.036). At 6 months post-treatment, 91.30% patients in the HSCT group and 8.78% patients in the non-HSCT group had achieved a normal blood profile (*P* < 0.0001). The time required to discontinue transfusions of red blood cells and platelets in the non-HSCT group was longer than in the HSCT group (*P* < 0.0001). Estimated overall survival (OS) at 6 years was similar in the two groups (75.5% ± 3.9% vs. 76.3% ± 3.1%, *P* = 0.996), while the estimated failure-free survival (FFS) at 6 years was 75.2% ± 3.8% in the HSCT group and 48.9% ± 3.7% in the non-HSCT group (*P* < 0.0001). Multivariate analysis showed that younger age, lower grade of infection (grade 1-2), and SAA (vs. very SAA) were favorable factors for OS (*P* < 0.05), and that the choice of HSCT and younger age were favorable factors for FFS (*P* < 0.0001).

**Conclusion:**

These results suggest that allo-HSCT has a better chance of a successful outcome than non-HSCT in SAA patients with an infection.

## Introduction

Severe aplastic anemia (SAA) is a life-threatening bone marrow disorder characterized by peripheral blood pancytopenia and a hypocellular marrow (1–3). In Europe and North America, the annual incidence of acquired aplastic anemia is approximately 2–3 cases per million individuals, although this figure is higher in East Asia (4). If left untreated, SAA is almost always fatal, with infections and/or hemorrhagic complications being the major causes of death (5). The standard specific treatment for a newly diagnosed patient with SAA is either allogeneic hematopoietic stem cell transplantation (allo-HSCT) from an HLA-matched related donor (MRD) or immunosuppressive therapy (IST) using a combination of anti-thymocyte immunoglobulin (ATG) and ciclosporin A (CsA). These two treatment options have resulted in improved long-term survival in SAA patients (4, 5). However, there are still many challenges in the management of these patients such as how to select a specific treatment in the presence of active infection?

The risk of infection is determined by the neutrophil numbers and function of a patient, and therefore patients with SAA are at risk of bacterial and fungal infections (6, 7). These infections have a very high mortality in SAA patients because of their prolonged periods of severe neutropenia (4). For SAA patients, attaining early neutrophil recovery is very important. Before a specific treatment is administered, it is essential to ensure that the patient does not have an active infection (4). It is dangerous to give ATG in the presence of an infection because they are an established adverse factor for outcomes and should be treated before starting IST (4, 8). However, in some cases it may be necessary to proceed with HSCT in the presence of an active infection because this therapy offers the best chance of early neutrophil recovery, while delaying the transplant may risk progression of the infection (8). HSCT in SAA patients with an infection is therefore regarded as “salvage therapy” or “salvage transplant”. Although allo-HSCT from an MRD is the preferred treatment, fewer than 30% of patients who require an allo-HSCT have an MRD (9, 10). For HSCT involving an unrelated donor (URD) it may take longer to identify a suitable donor. Haploidentical family donors HSCT (HID-HSCT) are available without delay, and as a result HID-HSCT has greatly increased as a treatment for SAA (11–16). It has been reported that patients who had a HID-HSCT as initial therapy had similar primary engraftment and survival outcomes to that observed in SAA patients who received MRD-HSCT (17). In China, alternative donor HSCT is recommended for newly diagnosed young SAA patients without an MRD (18).

As there are only a small number of studies that have investigated the outcomes of SAA patients with an infection who received an allo-HSCT, we are concerned about the mortality and graft failure in these patients. Xu et al. studied 65 SAA patients with an infection who received an allo-HSCT and reported encouraging results (19). However, this study did not investigate the outcomes of SAA patients with an infection who proceeded to an allo-HSCT compared with those who received non-transplantation therapy. The multicenter study described in this paper retrospectively reviewed 141 SAA patients with an infection who had an allo-HSCT as “first-line salvage therapy” and compared their results with 186 SAA patients with an infection who received non-transplantation therapy during the same time period.

## Patients and methods

### Patients

Between July 2004 and January 2020, 327 consecutive newly-diagnosed patients with SAA with an infection were enrolled in the study. The inclusion criteria for the study were: diagnosis and management of SAA or very SAA (VSAA) as defined by the guidelines (4); patients were younger than 65 years old; HSCT or non-transplantation treatment as an initial treatment; transfusion dependence (a hemoglobin < 60 × g/l or a platelet count of < 20 × 10^9^/l); the patients pressed for HSCT or non-transplantation treatment; and absence of severe liver, kidney, lung, and heart diseases. No patient enrolled in the study had a history of viral infection or exposure to drugs or other toxic agents. The exclusion criteria were: patients who were pregnant, diagnosed with other immunological diseases or had a positive test for myelodysplastic syndrome (MDS) based on bone marrow analyses. The mitomycin C-induced chromosomal breakage test was performed to exclude Fanconi anemia. Bone marrow cytogenetic analyses were performed in all the patients. Paroxysmal nocturnal hemoglobinuria (PNH) screening was performed in all patients by flow cytometry using anti-CD55 and anti-CD59 antibodies. For treatment of infections, empiric antibacterial agents such as piperacillin tazobactam, third-generation cephalosporins or carbapenems) were administered (20). If the fever persisted for more than three days, combined application of agents against gram-positive bacteria (vancomycin, linezolid, or teicoplanin) was considered (20). If fever persisted (4-7days) despite adequate antibacterial therapy, an antifungal therapy agent (amphotericin B, intravenous voriconazole, or caspofungin) was initiated (8, 20). If patients had a microbiologically documented infection, their therapy was modified according to susceptibility testing. All patients and donors provided written, informed consent for this treatment protocol. The patients provided written informed consent to participate in this study (if patients were younger than 18 years old, the consent was got from their parents).

### HLA typing and donor selection

HLA-A, -B, -C, DRB1, and -DQB1 typing of the recipients was performed. The donors were selected based on HLA typing, age, gender, health conditions, and willingness to donate. MRDs were the first-choice treatment. When an MRD was unavailable, alternative donors (unrelated donors (UDs), haploidentical donors, or unrelated umbilical cord blood (UCB) donors) were selected.

### Treatment protocol for HSCT

Patients with an MRD and UCB donors were treated according to the FLU/CY-based regimen which consisted of the following: Fludarabine (FLU), 30 mg/m^2^/day intravenously (i.v.) on day −7 to −2; cyclophosphoramide (CY), 50 mg/kg/day i.v. on day −4 to −3; and antithymocyte immunoglobulin (ATG, rabbit, Thymoglobuline^®^, Genzyme, Cambridge, MA, USA), 2.5 mg/kg/day i.v. on day −8 to −4, or antilymphocyte immunoglobulin (ALG, porcine, Wuhan Institute of Biological Products, China), 20 mg/kg/day i.v. on day −8 to −4, or anti-thymocyte globulin of Fresenius (ATG-F), 5 mg/kg/day i.v. on day −8 to −4. Patients with UDs or haploidentical donors were treated according to the BU/CY-based regimen which consisted of the following: Busulfan (Bu), 3.2 mg/kg/day i.v. on day −7 and −6; CY, 50 mg/kg/day i.v. on day −5 to −2; and ATG, 2.5 mg/kg/day i.v. on day −5 to −2, or ALG, 20 mg/kg/day i.v. on day −5 to −2, or anti-thymocyte globulin of Fresenius (ATG-F), 5 mg/kg/day i.v. on day −5 to −2.

The details of the stem cell mobilization, graft collection and infusion, graft versus host disease (GVHD) prophylaxis and treatment strategy, supportive care, and post-transplantation surveillance are consistent with those described in our previous papers (16, 21).

### Treatment protocol of non-HSCT

The patients with infection who had no fit donors or refused to undergo HSCT or could not receive HSCT were treated with anti-infection and supportive care therapy. This therapy was carried out in sterile rooms with strict reverse isolation from the beginning of therapy. After the infection was controlled, IST was started as follows: rabbit ATG (rATG) 3.5 mg/kg/day or porcine antihuman lymphocyte immunoglobulin (pALG) 30 mg/kg/day from day 1 to day 5, plus oral CsA treatment (3–5 mg/kg/day, with dose adjustments to achieve a whole-blood trough level of 200–250 ng/ml for adults and 150–200 ng/ml for children) or oral CsA alone. All the patients were treated with 5 μg/kg/day G-CSF SQ, with the dose of G-CSF decreased or stopped when the neutrophil count ≥ 2.0 × 10^9^/l (22).

### Definitions

After HSCT, the first time that the absolute neutrophil count (ANC) exceeded 0.5 × 10^9^/l for three consecutive days was defined as neutrophil engraftment. The first time the platelet count was > 20 × 10^9^/l without transfusion support for seven consecutive days was defined as platelet engraftment. Primary graft failure (GF) was defined as failure to achieve neutrophil engraftment after HSCT until day +28, while secondary graft failure was defined as the absence of graft function after achievement of initial full engraftment (23). Delayed platelet recovery was defined as platelet engraftment > 30 days (4).

A complete response (CR) was defined as transfusion independence associated with a hemoglobin level normal for age, a neutrophil count of > 1.5 × 10^9^/l, and a platelet count of > 150 × 10^9^/l. A partial response (PR) was defined as no longer meeting the criteria for SAA and no transfusion dependence for platelets or red blood cells. Transfusion dependence was classified as no response (NR) (4). Patients who died within 90 days of CsA or patients who underwent HSCT prior to 6 months were considered as non-responders (4, 24). Relapse was defined as the recurrence of disease.

Death without disease progression was defined as treatment-related mortality (TRM). Early mortality was defined as death within the first 60 days after HSCT or IST treatment. Failure-free survival (FFS) was defined as survival with a response. Death, no response by 6 months, disease progression requiring clinical intervention, and relapse were considered treatment failures for CsA, while death, engraftment failure, graft rejection, and relapse were considered treatment failures for HSCT. Infection was assessed per the NIH’s Common Toxicity Criteria for Adverse Events (CTCAE), Version 4.0 (25).

### Statistical analysis

The statistical analyses were conducted on data available from the date of treatment to the final date of patient follow-up (July 31, 2021). The patient characteristics were compared using the chi-square test and the nonparametric test for continuous variables. Fisher’s exact test was used when the number of subjects was less than 5 in any group. Based on clinical significance from previous evidence, we enrolled age, gender, grade of infection, treatment (HSCT or non-HSCT), and disease severity in the univariate analysis. The cumulative incidences of GVHD were estimated using the competing risk model, with death as the competing event. The probabilities of OS and FFS were estimated from the time of treatment using the Kaplan–Meier method, with comparisons of the different patient groups carried out using the log-rank test. For multivariate analysis, the Cox proportional hazard regression model was used to analyze OS and FFS. The statistical analyses were performed using SPSS version 22.0 (SPSS, Chicago, IL, U.S.A). All *P* values were two-sided and the results were considered statistically significant when *P* < 0.05.

## Results

### Patient characteristics

A total of 327 patients were enrolled in the study. Of these, 141 (43.12%) patients were enrolled in the HSCT group and 186 (56.88%) in the non-HSCT group. The characteristics of the patients and donors are shown in **Tables 1** and **Table 2**, respectively. There were no differences between the two groups for age and presence of the PNH clone (*P* > 0.05). The HSCT group had a higher proportion of males, VSAA and grade 3 infections than that observed in the non-HSCT group (*P* < 0.05), while the rate of grade 1 infection was lower in the HSCT group than in the non-HSCT group (*P* = 0.014).

**Table 1:** Characteristics of SAA patients and clinical outcomes after treatment.

Variable	HSCT (n=141)	Non-HSCT (n=186)	p-Value
Clinical characteristics			
Median age, years (range)	26 (1–58)	12 (1–65)	0.002
Gender (male/female)	90/51	95/91	0.021
Disease and status at therapy, no. (%)			<0.001
SAA	54 (38.30)	113 (60.75)	
VSAA	87 (61.70)	73 (39.25)	
SAA with PNH clone, no. (%)	5 (3.55)	1 (0.54)	0.111
ECOG score, median (range)	2 (1–3)	2 (1–3)	0.476
Median time from diagnosis to treatment, months (range)	1 (0.5–6)	1 (0.2–4)	0.251
Infection, no. (%)			
Pulmonary infections	87 (61.70)	74 (39.78)	<0.001
Septicemia	27 (19.15)	33 (17.74)	0.745
Febrile neutropenia	17 (12.06)	21 (11.29)	0.830
Soft tissue infection	24 (17.02)	17 (9.14)	0.033
Upper respiratory tract infection	5 (3.55)	36 (19.35)	<0.0001
Anal infection	2 (1.42)	7 (3.76)	0.346
Intestinal infection	2 (1.42)	13 (6.99)	0.017
Viremia	2 (1.42)	2 (1.08)	1.000
Intra-abdominal	0 (0.00)	2 (1.08)	0.508
Urinary tract infection	0 (0.00)	3 (1.61)	0.353
Sinusitis	2 (1.42)	2 (1.08)	1.000
Grade 1	20 (14.18)	48 (25.81)	0.010
Grade 2	35 (24.82)	47 (25.27)	0.927
Grade 3	81 (57.45)	77 (41.40)	0.004
Grade 4	5 (3.55)	14 (7.53)	0.128
Bacteria	52 (36.88)	71 (38.17)	0.811
Fungus	8 (5.67)	13 (6.99)	0.649
Virus	2 (1.42)	2 (1.08)	1.000
Uncertainty	79 (56.03)	100 (53.77)	0.684
Response to antibiotic treatment before HSCT or IST			
Control	32 (22.70)	156 (83.87)	<0.0001
Stable	102 (72.34)	0 (0.00)	<0.0001
Death	7 (4.96)	30 (16.13)	0.002
IST after the infection was controlled			
ATG/ALG + CsA	–	96 (51.61)	–
CsA	–	60 (32.26)	–
Median neutrophil count to reach ≥ l × 10^9^/L, days (range)	14 (10–27)	38 (2–84)	<0.0001
Median time no transfusion dependence for RBC, days (range)	22 (11–41)	72 (2–297)	<0.0001
Median time no transfusion dependence for plts, days (range)	14 (7–101)	69 (0–314)	<0.0001
Patients with normal blood routine at 6 months, no. (%)	105 (91.30)	13 (8.78)	<0.0001
Early death, no. (%)	15 (10.64)	26 (13.98)	0.366
Relapse, no. (%)	0 (0.00)	4 (4.82)	0.045
Secondary clonal disease, no. (%)	1 (0.71)	2 (1.08)	1.000
Causes of death, no. (%)			
Secondary graft failure	1 (3.23)	–	–
GVHD	7 (22.58)	–	–
Infection	19 (61.29)	29 (77.78)	0.317
Hemorrhage	0 (0.00)	11 (22.22)	0.004
PTLD	2 (6.45)	–	–
Multiple organ failure	1 (3.23)	0 (0.00)	0.437
MDS	1 (3.23)	0 (0.00)	0.437
Median follow-up time among living patients, months (range)	72 (16–140)	92 (12–150)	<0.0001

HSCT, hematopoietic stem cell transplantation; SAA, severe aplastic anemia; VSAA, very severe aplastic anemia; PNH, paroxysmal nocturnal haemoglobinuria; ECOG, eastern cooperative oncology group; IST, immunosuppressive therapy; ATG, anti-thymocyte immunoglobulin; ALG, antihuman lymphocyte immunoglobulin; CsA, ciclosporin A; RBC, red blood cell; GVHD, graft versus host disease; PTLD, post-transplantation lymphoproliferative diseases; MDS, myelodysplastic syndrome.

**Table 2:** Characteristics of SAA patients and donors in HSCT group.

Variable	N=141
Donor median age, years (range)	30 (9–63)
Type of donor, n (%)	
MRD	45 (31.91)
Haploidentical	78 (55.32)
UD	13 (9.22)
UCB	5 (3.55)
Donor–recipient sex match, non-UCB, no. (%)	
Male–male	54 (38.30)
Male–female	21 (14.90)
Female–male	33 (23.40)
Female–female	28 (19.86)
Donor–recipient relationship, no. (%)	
Mother–child	11 (7.80)
Father–child	27 (19.15)
Child–mother	6 (4.26)
Child–father	5 (3.55)
Siblings	74 (52.48)
Blood types of donor to recipient, non-UCB, no. (%)	
Matched	63 (44.68)
Major mismatched	23 (16.31)
Minor mismatched	32 (22.70)
Major and minor mismatched	18 (12.77)
Donor–recipient sex match, UCB, no. (%)	
Male–male	2 (1.42)
Male/female–female	2 (1.42)
Female–male	1 (0.71)
Blood types of donor to recipient, UCB, no. (%)	
B–O	2 (1.42)
O–B	1 (0.71)
O/B–O	1 (0.71)
AB–AB	1 (0.71)
Source of graft, no. (%)	
BM	3 (2.13)
PB	24 (17.02)
BM + PB	109 (77.30)
UCB	5 (3.55)
Incidences of infection after HSCT, no. (%)	
Bacterial	66 (46.81)
Fungal	17 (12.06)
CMV viremia	49 (34.75)
EBV viremia	31 (21.99)

SAA, severe aplastic anemia; HSCT, hematopoietic stem cell transplantation; MRD, matched related donor; UD, unrelated donor; UCB, unrelated cord blood; BM, bone marrow; PB, peripheral blood; MNC, mononuclear cell; CMV, cytomegalovirus; EBV, Epstein– Barr virus.

### Outcomes of HSCT

In the HSCT group, 129 of the 141 patients survived for more than 28 days, with a median mononuclear cell dose in the grafts of 10.90 (range, 0.23–26.01) × 10^8^/kg and a CD34^+^ cell dose of 4.23 (range, 0.15–11.00) × 10^6^/kg. Fifteen patients had an early death. Of the evaluable 133 patients, three patients experienced primary graft failure, while the remaining 130 patients achieved successful donor engraftment, although two patients experienced a secondary GF. Seven patients experienced GF of platelets and nine patients experienced delayed platelet engraftment. The median time to neutrophil engraftment was 11 days (range, 7–22), while the median time to platelet engraftment was 14 days (range, 7–101). The median time taken for the neutrophil count to reach ≥ l × 10^9^/l was 14 days (range, 10–27) and to discontinue the transfusion of RBCs and platelets was 22 days (range, 11–41) and 14 days (range, 7–101), respectively. Six months after HSCT, 105 of the 115 evaluable patients (91.30%) had achieved a normal blood profile (**Table 1**).

**Figure 1:**
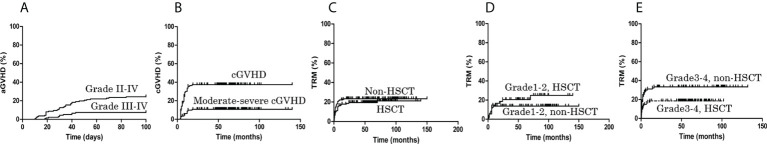
The cumulative incidence of graft versus host disease (GVHD) and treatment-related mortality (TRM). **(A)** In the allo-HSCT group, the cumulative incidence of grades II to IV acute GVHD (aGVHD) on day +100 was 24.27%, and the cumulative incidence of grades III and IV aGVHD on day +100 was 7.41%. **(B)** The cumulative incidence of chronic GVHD (cGVHD) was 37.40%, and the cumulative incidence of moderate–severe cGVHD was 11.34%. **(C)** During the follow-up period, TRM in the HSCT and non-HSCT groups was 21.13% and 23.27%, respectively (*P =* 0.464). **(D)** The TRM of grade 1-2 infection in the HSCT and non-HSCT groups was 24.99% and 13.68%, respectively (*P =* 0.206). **(E)** The TRM of grade 3-4 infection in the HSCT group was lower than non-HSCT group (18.54% vs. 33.33%, *P =* 0.036).

The cumulative incidence on day +100 of grades II to IV acute GVHD (aGVHD) was 24.27%, and for grades III and IV aGVHD was 7.41% (**Figure 1A**). The cumulative incidence of chronic GVHD (cGVHD) was 37.40%, while the cumulative incidence of moderate–severe cGVHD was 11.34% (**Figure 1B**). The cumulative incidence on day +100 of grades II to IV aGVHD in MRD-HSCT, URD-HSCT and HID-HSCT was 9.09%%, 25.00%, 31.58%; grades III and IV aGVHD was 2.33%, 8.33%, 10.53%. The cumulative incidence of cGVHD in MRD-HSCT, URD-HSCT and HID-HSCT was 21.76%, 40.00%, 47.69%%; the cumulative incidence of moderate–severe cGVHD was 11.08%, 11.11%, 13.85%.

### Outcomes of non-HSCT

Twenty-six patients experienced early death because of an infection, 97 patients received ATG/ALG + CsA treatment, and 59 patients received CsA treatment after the infection was controlled. The response to IST at 6 months showed 83 of the 148 evaluable patients (56.08%) improved with first-line IST and achieved either a complete response (*n* = 13, 8.78%) or partial response (*n* = 70, 47.30%). All these patients achieved transfusion independence. The median time taken for the neutrophil count to reach ≥ l × 10^9^/l for three consecutive days was 38 days (range, 2–84). The median times required to discontinue the transfusion of RBC and platelets were 72 days (range, 2–297) and 69 days (range, 0–314), respectively. These were longer than those observed in the HSCT group (*P* < 0.0001). Six months after treatment, 13 patients (8.78%) had achieved normal blood profiles (**Table 1**).

During follow-up, 4 cases showed NR at 6 months and therefore received salvage HSCT from either a MRD (*n* = 1), a haploidentical family donor (*n* = 2), or an UD (*n* = 1).

### Treatment-related mortality, relapse, and secondary clonal disease

During the follow-up period, TRM in the HSCT and non-HSCT groups was 21.13% and 23.27%, respectively (*P =* 0.464) (**Figure 1C**). The TRM of grade 1-2 infections in the HSCT and non-HSCT groups was 24.99% and 13.68%, respectively (*P =* 0.206) (**Figure 1D**), although for grade 3-4 infections the TRM in the HSCT group was lower than that in the non-HSCT group (18.54% vs. 33.33%, *P =* 0.036) (**Figure 1E**). No patient in the HSCT group relapsed whereas 4 (4.82%) responders in the non-HSCT group relapsed (*P* = 0.045). As shown in **Table 1**, one (0.71%) patient in the HSCT group developed secondary clonal disease (MDS), while in the non-HSCT group two (1.08%) responders developed secondary clonal disease (one PNH, one MDS) (*P =* 1.000).

### Survival

**Figure 2:**
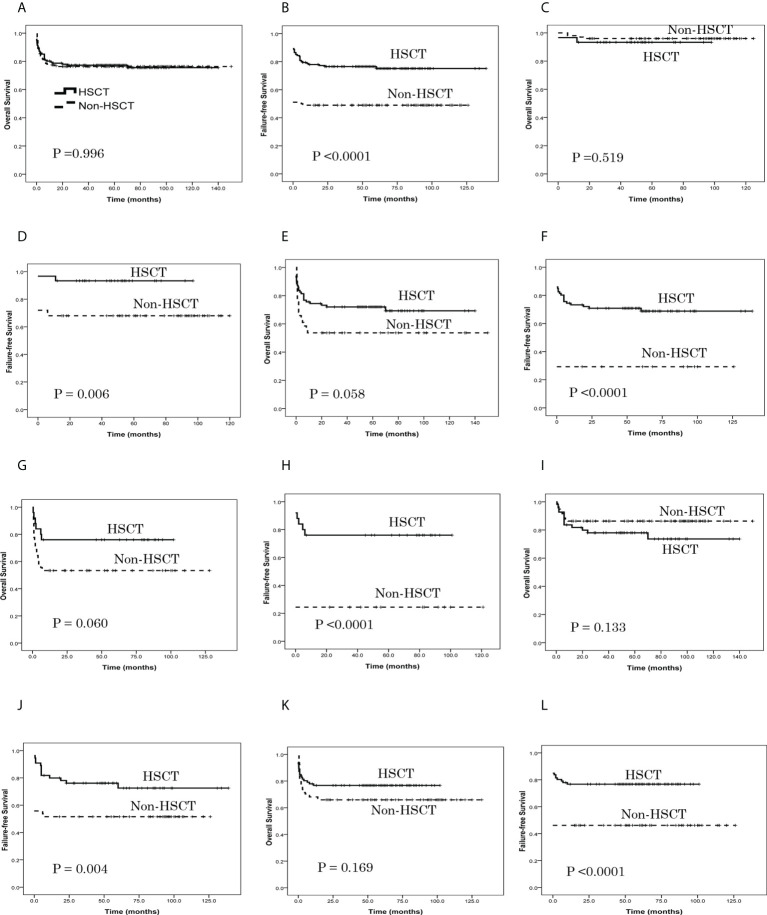
Patient overall survival (OS) and failure-free survival (FFS), as assessed using Kaplan-Meier analysis. **(A)** The estimated OS at 6 years was 75.5% ± 3.9% in the HSCT group and 76.3% ± 3.1% in the non-HSCT group (*P* = 0.996). **(B)** The estimated FFS at 6 years was 75.2% ± 3.8% in the HSCT group and 48.9% ± 3.7% in the non-HSCT group (*P* < 0.0001). **(C)** For patients aged ≤ 14 years, the estimated OS at 6 years was 93.3% ± 4.6% in the HSCT group and 96.0% ± 2.0% in the non-HSCT group (*P* = 0.519). **(D)** For patients aged ≤ 14 years, the estimated FFS at 6 years was 93.3% ± 4.6% in the HSCT group and 68.0% ± 4.7% in the non-HSCT group (*P* = 0.006). **(E)** For patients aged 15–39 years, the estimated OS at 6 years was 69.2% ± 5.4% in the HSCT group and 53.7% ± 7.8% in the non-HSCT group (*P* = 0.058). **(F)** For patients aged 15–39 years, the estimated FFS at 6 years was 68.6% ± 5.2% in the HSCT group and 29.3% ± 7.1% in the non-HSCT group (*P* < 0.0001). **(G)** For patients aged ≥ 40 years, the estimated OS at 6 years was 76.0% ± 8.5% in the HSCT group and 53.3% ± 7.4% in the non-HSCT group (*P* = 0.060). **(H)** For patients aged ≥ 40 years, the estimated FFS at 6 years was 76.0% ± 8.5% in the HSCT group and 24.4% ± 6.4% in the non-HSCT group (*P* < 0.0001). **(I)** For grade 1-2 infection, the estimated OS at 6 years was 73.6% ± 6.8% in the HSCT group and 86.3% ± 3.5% in the non-HSCT group (*P* = 0.133). **(J)** For grade 1-2 infection, the estimated FFS at 6 years was 72.5% ± 6.5% in the HSCT group and 51.6% ± 5.1% in the non-HSCT group (*P* = 0.004). **(K)** For grade 3-4 infection, the estimated OS at 6 years was 76.7% ± 4.6% in the HSCT group and 65.9% ± 5.0% in the non-HSCT group (*P* = 0.169). **(L)** For grade 3-4 infection, the estimated FFS at 6 years was 76.7% ± 4.6% in the HSCT group and 46.2% ± 5.2% in the non-HSCT group (*P* < 0.0001).

As shown in **Table 3**, the estimated OS at 6 years in the HSCT group and non-HSCT group was similar (*P* = 0.996) (**Figure 2A**), while the estimated FFS at 6 years in the HSCT group was higher than in the non-HSCT group (*P* < 0.0001) (**Figure 2B**). For patients aged ≤ 14 years, the estimated OS at 6 years in the HSCT group and non-HSCT group was similar (*P* = 0.519) (**Figure 2C**); the estimated FFS at 6 years in the HSCT group was higher than in the non-HSCT group (*P* = 0.006) (**Figure 2D**). For patients aged 15–39 years, there was no significant difference in the estimated OS at 6 years between the two groups (*P* = 0.058) (**Figure 2E**); the estimated FFS at 6 years in the HSCT group was higher than in the non-HSCT group (*P* < 0.0001) (**Figure 2F**). For patients aged ≥ 40 years, there was no significant difference in the estimated OS at 6 years between the two groups (*P* = 0.060) (**Figure 2G**); the estimated FFS at 6 years in the HSCT group was higher than in the non-HSCT group (*P* < 0.0001) (**Figure 2H**). For a grade 1-2 infection, there was no significant difference in the estimated OS at 6 years between the two groups (*P* = 0.133) (**Figure 2I**); the estimated FFS at 6 years in the HSCT group was higher than in the non-HSCT group (*P* = 0.004) (**Figure 2J**). For a grade 3-4 infection, there was no significant difference in the estimated OS at 6 years between the two groups (*P* = 0.169) (**Figure 2K**); the estimated FFS at 6 years in the HSCT group was higher than in the non-HSCT group (*P* < 0.0001) (**Figure 2L**).

**Table 3:** OS and FFS of SAA patients in HSCT group and non-HSCT group.

Variable	HSCT	Non-HSCT	p-Value
The estimated OS at 6 years	75.5% ± 3.9%	76.3% ± 3.1%	0.996
The estimated FFS at 6 years	75.2% ± 3.8%	48.9% ± 3.7%	< 0.0001
The estimated OS at 6 years for patients aged ≤ 14 years	93.3% ± 4.6%	96.0% ± 2.0%	0.519
The estimated FFS at 6 years for patients aged ≤ 14 years	93.3% ± 4.6%	68.0% ± 4.7%	0.006
The estimated OS at 6 years for patients aged 15-39 years	69.2% ± 5.4%	53.7% ± 7.8%	0.058
The estimated FFS at 6 years for patients aged 15-39 years	68.6% ± 5.2%	29.3% ± 7.1%	< 0.0001
The estimated OS at 6 years for patients aged ≥ 40 years	76.0% ± 8.5%	53.3% ± 7.4%	0.060
The estimated FFS at 6 years for patients aged ≥ 40 years	76.0% ± 8.5%	24.4% ± 6.4%	< 0.0001
The estimated OS at 6 years for grade 1-2 infection	73.6% ± 6.8%	86.3% ± 3.5%	0.133
The estimated FFS at 6 years for grade 1-2 infection	72.5% ± 6.5%	51.6% ± 5.1%	0.004
The estimated OS at 6 years for grade 3-4 infection	76.7% ± 4.6%	65.9% ± 5.0%	0.169
The estimated FFS at 6 years for grade 3-4 infection	76.7% ± 4.6%	46.2% ± 5.2%	< 0.0001

OS, overall survival; FFS, failure-free survival; SAA, severe aplastic anemia; HSCT, hematopoietic stem cell transplantation.

Before 2010, there were only 4 patients in the HSCT group and 9 patients in the non-HSCT group. So we compared the survival of the two groups from 2010 to 2015 and from 2016 to 2020. From 2010 to 2015, the estimated OS at 6 years was 73.2% ± 5.0% in the HSCT group and 81.6% ± 3.5% in the non-HSCT group (*P* = 0.203); the estimated FFS at 6 years was 73.6% ± 5.0% in the HSCT group and 56.0% ± 4.4% in the non-HSCT group (*P* = 0.007). From 2016 to 2020, the estimated OS at 6 years was 80.7% ± 5.2% in the HSCT group and 65.4% ± 6.6% in the non-HSCT group (*P* = 0.094); the estimated FFS at 6 years was 78.9% ± 5.4% in the HSCT group and 36.5% ± 6.7% in the non-HSCT group (*P* < 0.0001) (**Figures S1A, B**). In the HSCT group, the estimated OS at 6 years was 80.0% ± 6.0% for MRD-HSCT and 77.8% ± 4.7% for HID-HSCT (*P* = 0.948) (**Figure S1C**), while the estimated FFS at 6 years was 76.7% ± 6.6% for MRD-HSCT and 76.8% ± 4.8% for HID-HSCT (*P* = 0.838) (**Figure S1D**).

Multivariate analysis showed younger age, a lower grade of infection, and SAA (vs. VSAA) were favorable factors for OS (*P* < 0.05), while the choice of HSCT and younger age were favorable factors for FFS (*P* < 0.0001) (**Table 4**).

**Table 4:** Multivariate analysis of favorable factors associated with OS and FFS.

Outcome	Hazard ratio	95% CI	P
OS			
HSCT	0.786	0.493–1.253	0.312
Age	2.356	1.461–3.799	<0.001
Grade of infection	1.971	1.216–3.195	0.006
SAA (vs. VSAA)	1.785	1.107–2.877	0.017
FFS			
HSCT	0.426	0.284–0.638	<0.0001
Age	1.719	1.173–2.519	0.005
Grade of infection	1.144	0.801–1.634	0.460
SAA (vs. VSAA)	1.014	0.708–1.453	0.939

OS, overall survival; FFS, failure-free survival; HSCT, hematopoietic stem cell transplantation; SAA, severe aplastic anemia; VSAA, very severe aplastic anemia.

## Discussion

Infections remain the main cause of death for patients with SAA despite advances in IST (26). Treating SAA patients in the presence of an active infection is a challenge because infections have a very high mortality in these patients as they have prolonged periods of severe neutropenia. Attaining an early neutrophil recovery is very important in SAA patients with an infection, with the majority of patients not responding well to therapy even after receiving antibacterial/antifungal agents and G-CSF. Although IST using a combination of ATG and CsA have helped SAA patients to expect a long-term survival it is dangerous to administer IST in the presence of an infection and therefore the infection should be treated before giving IST (8). Otherwise, after IST the majority of responses for hematopoietic recovery usually take more than 3 months (24). In some cases it may be necessary to proceed with HSCT in the presence of an active infection because the transplant offers the best chance of early neutrophil recovery and delaying the transplant may risk progression of the fungal infection (8, 26). However, the presence of an infection is an adverse factor for outcome after HSCT and currently there is no information on the outcomes of SAA patients with an infection who receive an allo-HSCT compared with those who have non-transplantation therapy.

There are reports that infection may increase the incidence of graft rejection (24, 26). However, there were only few papers reported the treatment results of SAA patients with an infection. We were therefore concerned about mortality and graft failure for an allo-HSCT during the treatment of infection in AA patients. Xu et al. (19) reported the SAA patients with an infection in the anti-infection complete response (CR) who received an allo-HSCT had the similar outcomes with partial response/stable disease (PR/SD). The results indicated that the cumulative incidences of myeloid engraftment in the anti-infection PR/SD (n = 51) group and the CR (n = 14) group were 98% and 100%, respectively (*P* = 0.785). There were comparable results for 3-year estimated OS (85.4% vs. 92.9%; *P* = 0.530) and FFS (82.7% vs. 92.9%; *P* = 0.458) between the 2 groups. No differences were found in the cumulative incidences of platelet engraftment (96.1% vs. 100%; *P* = 0.613), grade II–IV aGVHD (23.5% vs. 14.3%; *P* = 0.466), grade III–IV aGVHD (3.9% vs. 7.1%; *P* = 0.598), cGVHD (27.1% vs. 14.3%; *P* = 0.358) or extensive cGVHD (4.1% vs. 0%; *P* = 0.442) between the PR/SD and CR groups. In a previous study we observed no difference in the median time for myeloid engraftment, median time for platelet engraftment, early mortality, graft failure, and survival between the infection and non-infection groups following a SAA HSCT (*P*>0.05) (27). For SAA patients with an infection, attaining early neutrophil recovery is very important. These infections have a very high mortality in SAA patients because of their prolonged periods of severe neutropenia. For IST, it is essential to ensure that the patient does not have an active infection. In the treatment period of infections before IST, a lot of patients died. These results indicated that allo-HSCT was a feasible therapeutic option for SAA patients with an infection.

In the current study, multivariate analysis, younger age, lighter grade of infection (grade 1-2), and SAA (vs. VSAA) were favorable factors for OS (*P* < 0.05), while the choice of HSCT was a favorable factor for FFS (*P* < 0.0001). In the HSCT group, the rates of VSAA and grade 3 infection were higher (*P* < 0.05) and the rate of grade 1 infection lower (*P* = 0.010) than in the non-HSCT group. Although the HSCT group had unfavorable factors, the patients in this group achieved a similar 6-year OS and better 6-year FFS than that observed in the non-HSCT group. Taken together, these results indicate that HSCT achieves better results than non-HSCT treatment, especially in older patients and those with a more serious grade of infection.

Although an allo-HSCT from a HLA-identical sibling donor is the preferred initial treatment, fewer patients who required an allo-HSCT had a MRD, and therefore a HID-HSCT was carried out as a donor was available without delay. In this study, we found that patients who underwent HID-HSCT as initial therapy had similar OS and FFS to SAA patients who received a MRD-HSCT. A HID-HSCT should therefore be considered for SAA patients with an infection without an MRD because a HID-HSCT has the advantage of a donor being available without delay. Fortunately, eltrombopag (EPAG), an oral synthetic small-molecule thrombopoietin receptor agonist, was found to be effective in SAA patients (28, 29). Subsequent studies reported that combination therapy of IST and EPAG (IST+EPAG) achieved encouraging results in newly diagnosed SAA patients (30). The efficacy of IST in combination with eltrombopag for SAA patients with infection should be addressed in the future.

In conclusion, the encouraging results obtained in this study suggest that (i) SAA patients with infection who receive an allo-HSCT obtain better results than that observed with non-HSCT treatment, especially in older patients and those with a serious grade of infection; (ii) the speed of efficacy in the allo-HSCT group was faster than in the non-HSCT group. However, it is important to note that our study was limited by its retrospective design. The center effect should be considered in the present study. Although the identical inclusion and exclusion criteria of patients, there may still be some variation in different centers. Further well-designed, prospective, controlled multicenter cooperative studies are therefore needed to validate our results and to confirm the superiority of this approach.

## Data availability statement

The raw data supporting the conclusions of this article will be made available by the authors, without undue reservation.

## Ethics statement

Written informed consent from the patients/participants OR patients/participants legal guardian/next of kin was obtained to participate in this study in accordance with the national legislation and the institutional requirements.

## Author contributions

LL, LZ and DW designed the research; LL, MM and HH analysed the data and wrote the manuscript; and all authors provided patient data and gave final approval for the manuscript.

## Funding

This work was partially supported by grants from the National Key R&D Program of China (2016YFC0902800, 2017YFA0104502, and 2017ZX09304021), the Innovation Capability Development Project of Jiangsu Province (BM2015004), the Jiangsu Provincial Key Medical Center (YXZXA2016002), the Jiangsu Medical Outstanding Talents Project (JCRCA2016002), the Natural Science Foundation of Jiangsu Province (Grants No.BK20180202), the Priority Academic Program Development of Jiangsu Higher Education Institutions (PAPD), the Science Foundation of Suzhou (No.SKY2021040) and the Open Project of Jiangsu Biobank of Clinical Resources (No. SBK202003003).

## Acknowledgments

The authors would like to express their gratitude to EditSprings (https://www.editsprings.cn/) for the expert linguistic services provided.

## Conflict of interest

The authors declare that the research was conducted in the absence of any commercial or financial relationships that could be construed as a potential conflict of interest.

## Publisher’s note

All claims expressed in this article are solely those of the authors and do not necessarily represent those of their affiliated organizations, or those of the publisher, the editors and the reviewers. Any product that may be evaluated in this article, or claim that may be made by its manufacturer, is not guaranteed or endorsed by the publisher.

## Supplementary material

Supplementary Figure 1 Patient overall survival (OS) and failure-free survival (FFS), as assessed using Kaplan-Meier analysis. **(A)** From 2010 to 2015, the estimated OS at 6 years was 73.2% ± 5.0% in the HSCT group and 81.6% ± 3.5% in the non-HSCT group (*P* = 0.203); from 2016 to 2020, the estimated OS at 6 years was 80.7% ± 5.2% in the HSCT group and 65.4% ± 6.6% in the non-HSCT group (*P* = 0.094). **(B)** From 2010 to 2015, the estimated FFS at 6 years was 73.6% ± 5.0% in the HSCT group and 56.0% ± 4.4% in the non-HSCT group (*P* = 0.007); from 2016 to 2020, the estimated FFS at 6 years was 78.9% ± 5.4% in the HSCT group and 36.5% ± 6.7% in the non-HSCT group (*P* < 0.0001). **(C)** In the HSCT group, the estimated OS at 6 years was 80.0% ± 6.0% for MRD-HSCT and 77.8% ± 4.7% for HID-HSCT (*P* = 0.948). **(D)** In the HSCT group, he estimated FFS at 6 years was 76.7% ± 6.6% for MRD-HSCT and 76.8% ± 4.8% for HID-HSCT (*P* = 0.838).
