# Supplementary figures and images for: Severe aplastic anemia patients with infection who received an allogeneic hematopoietic stem cell transplantation had a better chance: Long-term outcomes of a multicenter study
Source: Front Immunol. 2022 Sep 5;13:955095. doi: 10.3389/fimmu.2022.955095 (PMC9483095; doi:10.3389/fimmu.2022.955095)

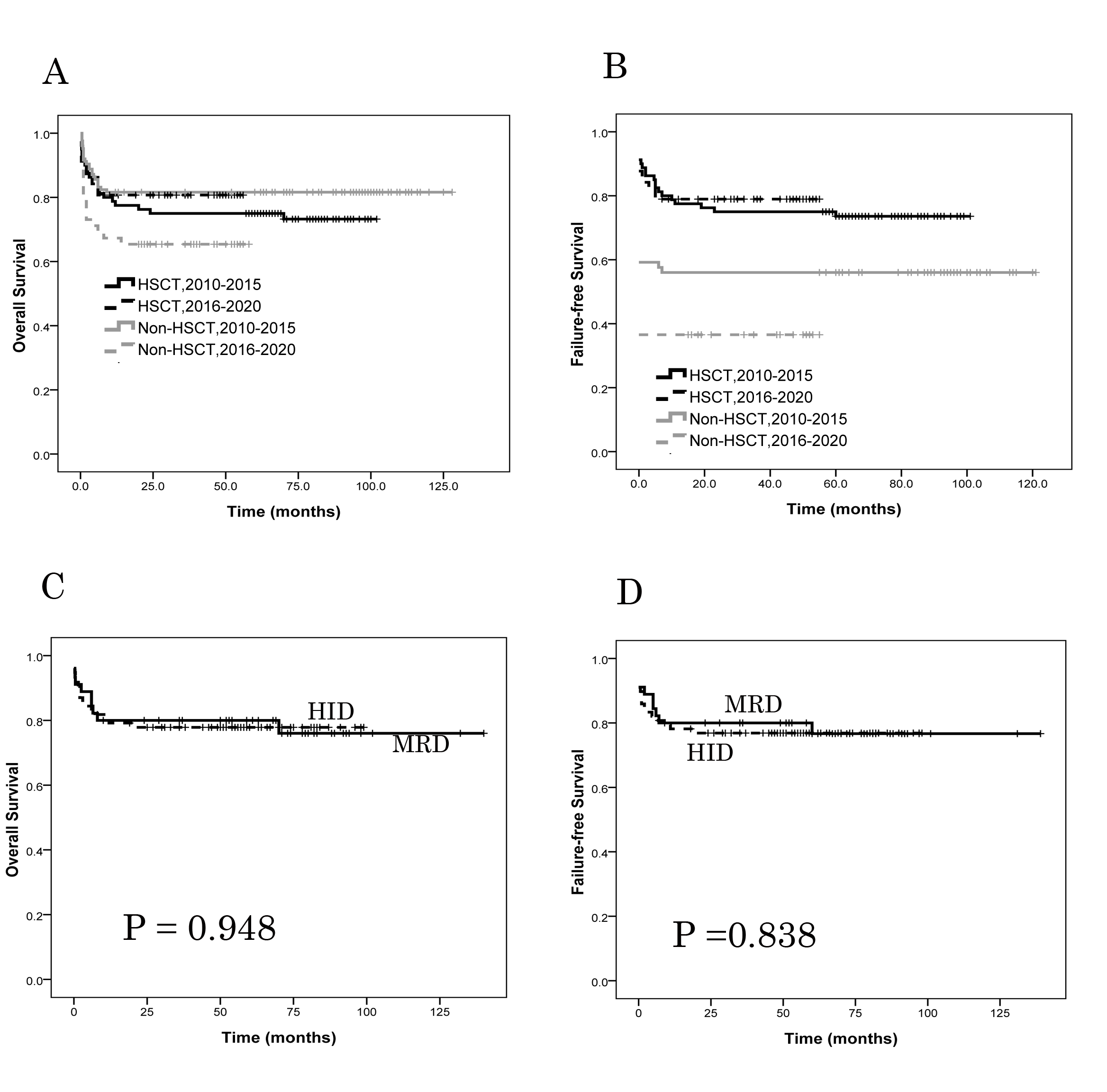

Supplement: Supplementary Figure 1 — Patient overall survival (OS) and failure-free survival (FFS), as assessed using Kaplan-Meier analysis. (A) From 2010 to 2015, the estimated OS at 6 years was 73.2% ± 5.0% in the HSCT group and 81.6% ± 3.5% in the non-HSCT group (P = 0.203); from 2016 to 2020, the estimated OS at 6 years was 80.7% ± 5.2% in the HSCT group and 65.4% ± 6.6% in the non-HSCT group (P = 0.094). (B) From 2010 to 2015, the estimated FFS at 6 years was 73.6% ± 5.0% in the HSCT group and 56.0% ± 4.4% in the non-HSCT group (P = 0.007); from 2016 to 2020, the estimated FFS at 6 years was 78.9% ± 5.4% in the HSCT group and 36.5% ± 6.7% in the non-HSCT group (P < 0.0001). (C) In the HSCT group, the estimated OS at 6 years was 80.0% ± 6.0% for MRD-HSCT and 77.8% ± 4.7% for HID-HSCT (P = 0.948). (D) In the HSCT group, he estimated FFS at 6 years was 76.7% ± 6.6% for MRD-HSCT and 76.8% ± 4.8% for HID-HSCT (P = 0.838). [file Image_1.tif]
